# Bach2 deficiency leads autoreactive B cells to produce IgG autoantibodies and induce lupus through a T cell-dependent extrafollicular pathway

**DOI:** 10.1038/s12276-019-0352-x

**Published:** 2019-12-09

**Authors:** Eunkyeong Jang, Un Kyo Kim, Kiseok Jang, Young Soo Song, Ji-Young Cha, Hansol Yi, Jeehee Youn

**Affiliations:** 1 Laboratory of Autoimmunology, Department of Anatomy and Cell Biology, College of Medicine, Hanyang University, Seoul, 04763 Korea; 2 Department of Pathology, College of Medicine, Hanyang University, Seoul, 04763 Korea; 3 Department of Biochemistry, Lee Gil Ya Cancer and Diabetes Institute, Gachon University, Incheon, 21936 Korea

**Keywords:** Autoimmunity, Autoimmune diseases

## Abstract

Class-switched IgG autoantibodies but not unswitched IgM autoantibodies play a crucial role in the development of systemic lupus erythematosus (SLE). Bach2 is known to be essential for class switch recombination of Ig genes, but recent genomic and clinical studies have suggested an association of Bach2 deficiency with SLE. This study was undertaken to examine the mechanism by which Bach2 regulates the development of SLE. Despite defects in Ig class switch recombination and germinal center formation when actively immunized, *Bach2*^*−/−*^ mice spontaneously accumulated IgG autoantibody-secreting cells without germinal center reactions in a regulatory T cell-independent manner, and this phenomenon was accompanied by manifestations akin to SLE. Transcriptome analyses revealed that Bach2 regulated the expression of genes related to germinal center formation and SLE pathogenesis in B cells. B cell-specific deletion of Bach2 was sufficient to impair the development of germinal center B cells but insufficient to promote the production of IgG autoantibodies. Bach2 deficiency caused CD4^+^ T cells to overexpress Icos and differentiate into extrafollicular helper T cells in a cell-autonomous manner. These findings suggest that Bach2-deficient autoreactive B cells preferentially react at extrafollicular sites to give rise to IgG class-switched pathogenic plasma cells and that this effect requires the help of Bach2^-^Icos^hi^ helper T cells. Thus, the cell-autonomous roles of Bach2 in B cells and in their cognate CD4^+^ T cells are required to maintain self-tolerance against SLE.

## Autoimmune disease: Bach2 orchestrates immune function

Bach2, a protein that regulates gene expression, is required in the B cells and T cells of the immune system to protect against autoimmune disease. Bach2 deficiency has previously been associated with systemic lupus erythematosus (SLE), but the mechanisms through which it contributes to the development of an immune response against healthy tissue in many parts of the body were unclear. Jeehee Youn at Hanyang University in Seoul, South Korea, and colleagues showed that B cells from mice lacking Bach2 produce self-reactive antibodies and express SLE-related genes. Furthermore, when they specifically deleted Bach2 in T cells, they found that it triggered differentiation into a type of T cell which promoted the maturation of self-reactive B cells. The authors conclude that Bach2 activity in both B cells and T cells is key to maintaining immune self-tolerance.

## Introduction

Systemic lupus erythematosus (SLE or lupus) is a systemic autoimmune disease characterized by an abundance of antibodies (Abs) against nuclear self-antigens^1^. Among the diverse isotypes of anti-nuclear Abs (ANAs), only IgG class-switched ANAs are considered pathogenic since they are deposited in tissues as immune complexes, leading to inflammation and end-stage organ damage in the kidneys, skin, and additional organs. In contrast, IgM class-unswitched ANAs elicit protective effects against autoimmunity by assisting in the clearance of cellular debris and interfering with the responses mediated by IgG ANAs^2–4^. There is little evidence for the pathogenicity of other isotypes, such as IgA and IgE. Therefore, the execution of IgG class switch recombination (CSR) prior to the emergence of Ab-secreting cells appears to be crucial for the development of SLE.

Although there are models in which T cell-independent Ab responses contribute to lupus in mice^5^, the majority of studies with lupus-prone mice and SLE patients indicate that T cell-dependent Ab responses are the main drivers of the disease. The initial interaction of antigen-engaged B cells with cognate CD4^+^ T cells at the border of the T cell zone and the follicle drives B cells to proliferate and diverge into extrafollicular (EF) plasma cells and germinal center (GC) B cells^6–8^. The EF and GC responses are instructed by specialized subsets of helper T (Th) effector cells, known as EF Th (Tefh) and follicular Th (Tfh) cells, respectively^9,10^. Despite some common phenotypes (Bcl6^+^Icos^+^CD40L^+^PSGL^lo^) and common mechanisms of B cell help, differences in chemokine receptors (CXCR5^+^CCR7^−^ for Tfh cells versus CXCR4^+^CCR7^+^ for Tefh cells) lead them to diverge into two anatomically distinct compartments. Nevertheless, it remains unclear what mechanisms control the B cell fate decision at the bifurcation point.

According to the traditional paradigm, plasma cells secreting class-switched and affinity-matured Abs develop mostly as a result of GC reactions^11–13^; hence, the GC has been emphasized as the main driver of SLE. However, this paradigm is based mainly on studies using acute immunization models and, for several reasons, may not be applicable to chronic autoimmune responses with a stochastic onset. First, self-reactive B cells tend to be excluded at the entrance of the follicle and may thus be preferentially activated to produce pathogenic IgG autoantibodies outside the follicle^14,15^. Second, some B cells undergo Ig CSR through the EF pathway^6,7^. Finally, the EF pathway is also implicated in SLE in some models^15–17^. These findings suggest that EF Ab responses are also important in the development of SLE. Hence, clarifying the mechanisms and genetic risk alleles of the EF pathway should provide insight into the pathogenesis of SLE.

Recent genome-wide association studies (GWASs) suggest that polymorphism of *BACH2* is associated with SLE^18,19^. This possibility is supported by a clinical study showing compromised expression of Bach2 in patients with SLE^20^. Bach2 is a transcription repressor with a basic region leucine zipper domain^18^. It forms heterodimers with small Maf proteins and binds to the Maf-recognition element (MARE) of target genes. Bach2 was initially identified as a B cell-specific factor required for CSR and somatic hypermutation (SHM) of Ig-encoding genes^21^. This activity can be explained in the context of the genetic regulatory network operating in B cells: Bach2 represses the expression of *Prdm1*, which encodes Blimp-1, a master regulator of plasma cell development; this delays induction of Blimp-1, thereby securing a time window for expression of *Aicda*, which encodes activation-induced cytidine deaminase (AID), an enzyme essential for both CSR and SHM, ultimately preventing the emergence of unswitched premature plasma cells^22^. In addition to its function in B cells, Bach2 participates in T cell-mediated immune responses in a cell-autonomous manner, as proven by skewed differentiation of CD4^+^ T cells toward inflammatory effector T cells (especially Th2 cells) at the expense of Foxp3^+^ regulatory T (Treg) cells in Bach2-deficient mice^23–25^. Although these findings establish Bach2 as a key regulator of immune resistance to the activities of inflammatory effector T cells, it is not clear whether Bach2 also affects the development of humoral effector Th cells.

Based on the evidence from human GWASs and the aforementioned clinical study^18–20^, one could anticipate that genetic deficiency in Bach2 would predispose individuals to SLE. However, this hypothesis would not explain how Bach2-deficient B cells, which are defective in CSR, are able to cause SLE, which is strongly dependent on IgG autoantibodies. Nor is it clear whether the cell-autonomous effects of Bach2 in B cells, in T cells, or in both T and B cells are important in the context of SLE. To answer these questions, we first examined whether Bach2-deficient mice spontaneously generate autoimmune responses accompanied by lupus. Surprisingly, we found that contradicting previous reports, Bach2-deficient autoreactive B cells gave rise to plasma cells producing IgG-switched autoantibodies, and this activity was sufficient to drive renal manifestations reminiscent of lupus. Using bone marrow (BM) reconstitution systems, we then dissected the cell-autonomous functions of Bach2 in B and T cells. Our findings provide evidence that genetic deficiency in Bach2 predisposes individuals to SLE via T cell-dependent EF activation of Ab responses.

## Materials and methods

### Mice

C57BL/6 mice congenic for *Bach2*^*+/−*^ originally donated by Dr. Kazuhiko Igarashi (Tohoku University, Sendai, Japan)^21^ were bred in the animal facility at Hanyang University under specific pathogen-free conditions. *Bach2*^*−/−*^ mice and their sex-matched littermates were used. CD45.1, *Rag2*^*−/−*^*, Tcrβ*^*−/−*^ and μMT (B cell-deficient) mice were obtained from Jackson Laboratory. All procedures were approved by the Institutional Animal Care and Use Committee, and all animal experiments were carried out in strict accordance with guidelines and regulations.

### Histopathologic examination

Mouse kidney tissues were examined by standard histopathologic methods as described^26^. To obtain histopathologic scores, more than 50 glomeruli per mouse were individually examined by a certified pathologist who was blinded to the sample genotypes.

### Fluorescence microscopy

Mouse kidneys and spleens were assayed *post mortem* by fluorescence immunohistochemical methods as described^27^. Frozen sections were stained with appropriate combinations of anti-B220-allophycocyanin (eBioscience), anti-GL7-FITC (BD Biosciences), anti-IgG-biotin (Sigma-Aldrich), and anti-IgM-biotin (Southern Biotech) Abs and streptavidin. GCs were counted at a magnification of X200, and glomerular Ig deposits were scored as mean fluorescence intensities using ImageJ software (NIH).

### Bone marrow reconstitution

*Rag2*^*−/−*^ mice were given 500 rad of total body γ-radiation and intravenously injected with 5 × 10^6^ cells of 3:1 mixtures of either *Bach2*^−/−^ or wild-type (WT) and either *Tcrβ*^*−/−*^ or μMT BM cells. They were treated with antibiotics (Baytril) for 2 weeks and assayed *ex vivo* 10-15 weeks post-transplant.

### Treg cell reconstitution

CD4^+^CD25^hi^ Treg cells of >98% purity were isolated from WT spleens with MACS columns (Miltenyi Biotec) followed by FACSaria III (BD Biosciences). The Treg cells were injected intravenously into approximately 8-week-old *Bach2*^*−/*^^−^ mice at 2–3 × 10^6^ cells/mouse and assayed *ex vivo* 4 or 8 weeks later.

### Retrovirus-transduced cell transfer

PLAT-E retroviral packaging cells were cotransfected with either MigR1–Bach2^28^ or empty vector and pCL–eco, and culture supernatants containing retroviruses were collected, as described^29^. CD45.1^+^CD4^+^ T cells from CD45.1^+^ mice negatively selected using EasySep (StemCell) were preactivated and spin-infected with retrovirus supernatants. GFP^+^ cells were sorted and transferred intravenously into *Tcrβ*^*−/−*^ mice at 5 × 10^5^ cells/mouse.

### Flow cytometry

Single-cell suspensions of spleens were prepared and assayed by flow cytometry as described^30^. The fluorochrome-conjugated monoclonal Abs used are listed in Supplementary Table 1. To detect cytokine expression, splenocytes were stimulated with 20 ng/ml phorbol 12-myristate 13-acetate (PMA) and 1 μM ionomycin (Sigma-Aldrich) in the presence of Golgi-stop reagent (BD Biosciences) for 5 h and treated with Cytofix/Cytoperm Fixation/Permeabilization Solution (BD Biosciences).

### ELISA and ELISPOT assay

Serum titers of anti-dsDNA and anti-muscarinic receptor 3 (M3R) Abs were determined as described previously^26^. Concentrations of total IgG and total IgM Abs were measured using ELISA kits purchased from Alpha Diagnostic International. To measure 4-hydroxy-3-nitrophenylacetyl (NP)-specific Ab, plates were coated with 10 μg/ml NP-BSA (Biosearch Technologies) and processed by standard ELISA methods. Anti-dsDNA and anti-M3R Ab-secreting cells were enumerated by ELISPOT assays as described^26^.

### Quantitative RT-PCR

Total RNA was extracted from cells and assayed by quantitative RT-PCR as described previously^28^. Levels of mRNA were quantified using the cycle threshold method (∆Ct) and normalized to β-actin mRNA levels. The primer sequences used are given in Supplementary Table 2.

### RNA sequencing (RNAseq) and transcriptome analysis

Total RNA was extracted from CD19^+^ B cells of Bach2 knockout (KO) and WT mice. cDNA libraries were prepared using a TruSeq Stranded mRNA LT Sample Prep kit (Illumina) and sequenced on a NovaSeq 6000 platform using 101 bp paired-read chemistry. Raw sequencing reads were subjected to quality control using FastQC, followed by read trimming with Trimmomatic. The resulting high-quality reads were mapped to the reference mouse genome (UCSC mm 10) via HISAT2. Read counts per gene were obtained by StringTie, using RefSeq_2017_06_12 as a reference. To generate a hierarchically clustered heatmap, count data were log-transformed with regularization and examined for differentially expressed genes (DEGs) using the Bioconductor pheatmap package. To identify DEGs, we used DESeq2, which operates based on a negative binomial distribution. Only protein-coding genes with nonzero read counts in all four samples were selected for further DEG analysis (12545 genes retained). The criteria for DEGs were defined as fold change (FC) > 2 and adjusted *P* value < 0.05 by Benjamini-Hochberg methods^31^. For unbiased identification of enriched pathways associated with transcriptomic changes, gene set enrichment analysis (GSEA) of the 186 MSigDB-based (v6.2) KEGG pathways was performed using log_2_FC as a ranking metric.

### Luciferase assay

A reporter construct containing 2000 bp of the human *ICOS* promoter upstream of the transcription start site was generated by PCR and cloned into the pGL4b vector (Promega). Jurkat T cells were transiently cotransfected with the reporter plus MigR1–Bach2 or empty vector and cultured in the presence or absence of PMA and ionomycin, and luciferase activity was assayed as described^28^.

### Chromatin immunoprecipitation–quantitative PCR (ChIP-qPCR)

Jurkat T cells were transfected with a FLAG-tagged Bach2 cDNA construct^28^, cultured in the presence or absence of PMA and ionomycin, and assayed as described^28^. Anti-FLAG Ab (F1804; Sigma-Aldrich) and control IgG (Sc-2027; Santa Cruz) were used to precipitate chromatin fragments. The primer sequences used are given in Supplementary Table 2.

## Results

### Bach2-deficient mice have elevated titers of IgG-switched autoantibodies

**Fig. 1:**
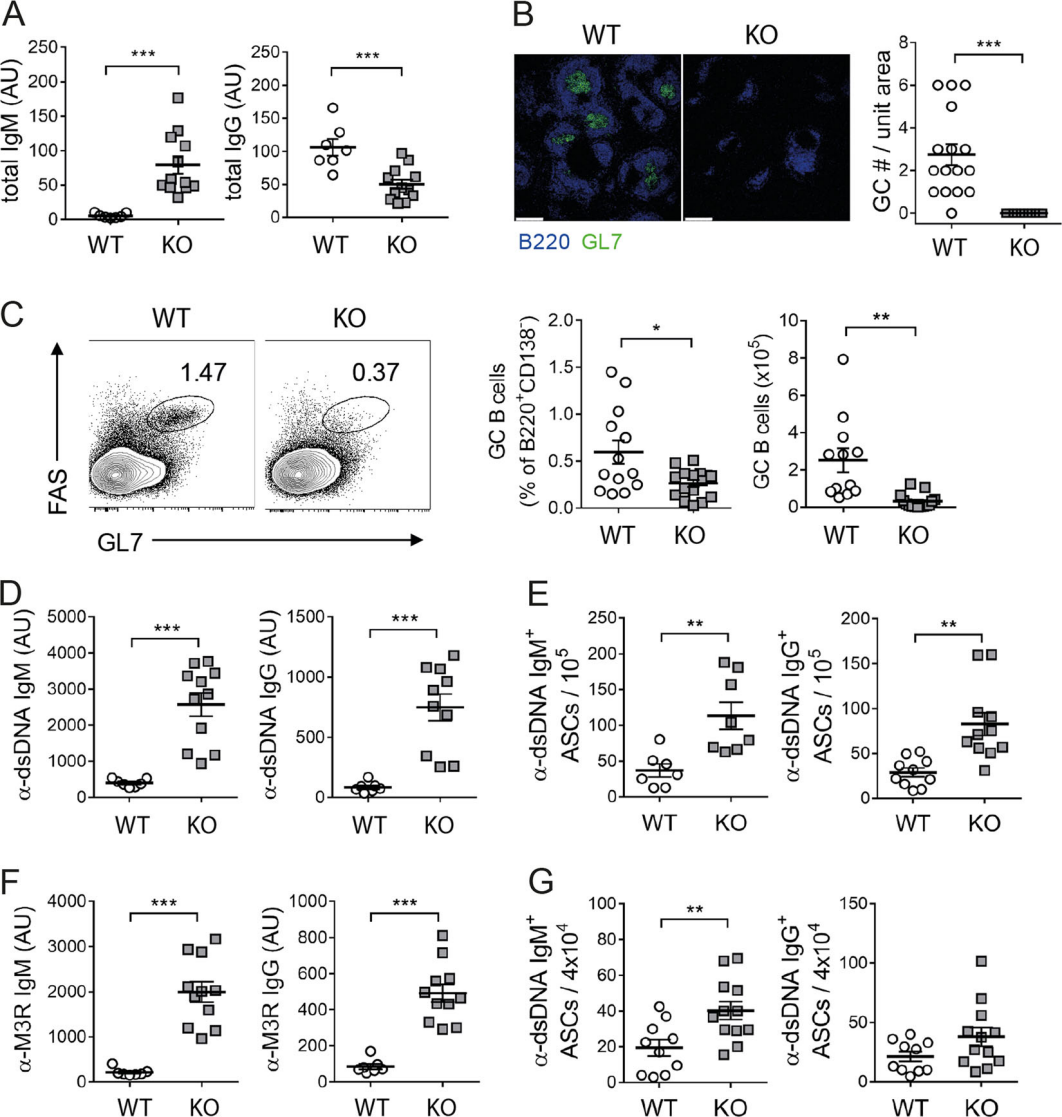
Features of autoantibodies and autoantibody-secreting cells in Bach2 KO mice. The serum and spleen were collected from approximately 20-week-old Bach2 KO mice and their WT littermates. Serum was assayed by ELISA to determine titers of total IgM and IgG **a**, anti-dsDNA IgM and IgG **d**, and anti-M3R IgM and IgG **f**. Spleens were examined by fluorescence confocal microscopy to detect GCs **b**, FACS to detect GC B cells **c**, and ELISPOT to count Ab-secreting cells **e**, **g**. Data are presented as the means ± SEMs in arbitrary units (AU) and Ab-secreting cells (ASCs) per indicated number of cells, pooled from three independent experiments. FACS profiles gated on B220^+^CD138^-^ cells are representative of three independent experiments. Scale bar, 250 μm. *p < 0.05, **p < 0.01 and ***p < 0.001 by Student’s *t*-test.

Bach2-deficient mice are reported to have a defect in GC formation, with reduced production of class-switched Abs, when challenged with exogenous antigens^21^. We confirmed that the absence of Bach2 impairs the production of IgG-switched Abs in B cells in response to deliberate immunization with a T cell-dependent eliciting antigen and even in the presence of appropriate signals in vitro (Supplementary Fig. 1). Since these results, along with those in previous investigations, were obtained using acute immunization models and in vitro settings, it was not clear whether they applied to chronic spontaneous immune activation arising at steady state. To clarify this matter, we examined Ab responses in untreated Bach2-deficient mice aged approximately 20 weeks. Although the serum titer of whole IgM was elevated in these mice, the serum titer of whole IgG was significantly reduced, indicative of impaired CSR (Fig. 1a). The Bach2-deficient mice did not form any GCs in their spleens, while substantial numbers of GCs were present in their WT littermates (Fig. 1b). In agreement with this result, B220^+^CD138^−^GL7^+^Fas^+^ GC B cells were significantly less numerous in the Bach2-deficient spleens than in those of WT mice (Fig. 1c). Thus, these results demonstrated that as in their responses to deliberate immunization, aged Bach2-deficient mice are defective in spontaneous GC formation, with reduced production of IgG-switched Abs.

Given the different molecular requirements for autoimmunity versus active immunization^32,33^, it was possible that Bach2 deficiency might not affect Ab responses against self-antigens in the same was as those against exogenous antigens. To address this possibility, we examined the titers and isotypes of anti-dsDNA Ab, a signature autoantibody for lupus, in aged Bach2-deficient mice. Surprisingly, they contained a significantly higher titer of IgG-switched Abs specific for dsDNA than their WT littermates (Fig. 1d). The fold difference was comparable to that of anti-dsDNA IgM titers. Consistent with this result, cells secreting anti-dsDNA IgG as well as those secreting anti-dsDNA IgM were more abundant in the spleens of the Bach2-deficient mice than in those of their WT littermates (Fig. 1e). We also found that another type of autoantibody to M3R protein^34^ behaved in the same was as anti-dsDNA Ab in terms of the pattern of CSR and tissue distribution of Ab-secreting cells (Fig. 1f, g). Thus, these results demonstrate that Bach2 deficiency stimulates the production of IgG-switched autoantibody-secreting cells in a GC-independent manner, and this activity does not occur in the case of deliberate immunization.

### Bach2-deficient mice exhibit histopathologic manifestations of lupus nephritis in a Treg cell-independent manner

**Fig. 2:**
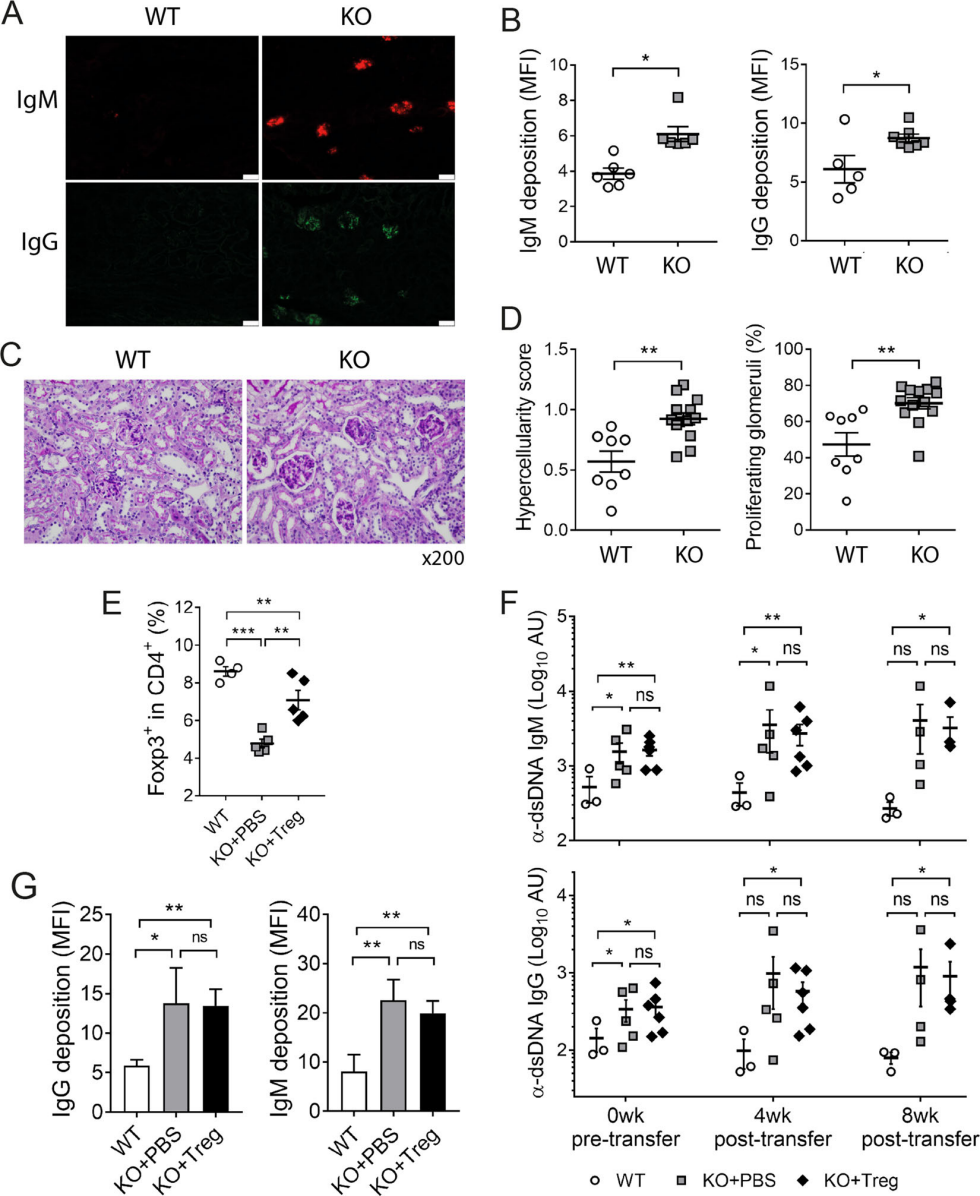
Histopathologic manifestations of lupus nephritis and the effect of Treg reconstitution in Bach2 KO mice. **a**–**d** Kidneys from approximately 20-week-old Bach2 KO mice and their WT littermates were examined by histopathologic methods. Representative immunohistochemical images of kidney sections stained with anti-mouse IgM (upper figures) and IgG (lower figures) Abs and mean fluorescence intensities (MFIs) **a**, **b**. Scale bar, 50 μm. Representative images stained with periodic acid-Schiff and histopathologic indexes **c**, **d**. **e**–**g** Bach2 KO and WT mice were infused intravenously with normal syngeneic Foxp3^+^ Treg cells or PBS as a vehicle control. Foxp3^+^CD4^+^ cells among spleen cells were examined 8 weeks after infusion **e**. Sera were collected from the recipients at the indicated times and assayed by ELISA **f**. All graphs display means ± SEMs, with symbols representing the values of individual mice. Kidney sections stained with anti-IgM or anti-IgG Abs were examined by confocal fluorescence microscopy and displayed as the mean ± SEM MFI **g**. *p < 0.05, **p < 0.01 and ***p < 0.001 by Student’s *t*-test. ns, not significant.

ANAs, especially anti-dsDNA IgG, are deposited in glomeruli in the form of immune complexes, leading to type III hypersensitivity-driven glomerulonephritis. To determine whether this process takes place in aged Bach2-deficient mice, we histopathologically examined their kidneys. As expected, deposits of IgG as well as IgM were more numerous in the glomeruli of Bach2-deficient mice than in those of WT controls (Fig. 2a, b). The Bach2-deficient mice also had elevated histopathologic indexes of lupus nephritis, as judged by their hypercellularity score and the percentage of proliferating glomeruli (Fig. 2c, d). Thus, the IgG autoantibodies that are enriched in Bach2-deficient mice appear to be sufficient to drive lupus nephritis.

In addition to its role as a B cell-specific regulator, Bach2 acts in a cell-autonomous manner to stabilize Foxp3^+^ Treg cells. Indeed, we observed that the proportion of Treg cells among CD4^+^ T cells was significantly reduced in Bach2-deficient mice. To determine whether the lupus-inducing autoimmunity in Bach2-deficient mice was simply due to this Treg cell deficiency, we adoptively transferred normal Treg cells into Bach2 KO mice and were able to partially but significantly restore their Treg cell population (Fig. 2e). Treg cells harvested from the Treg cell recipients suppressed the proliferation of conventional T cells as efficiently as those from PBS recipients (Supplementary Fig. 2a). In addition, the lung inflammation prominent in PBS-infused Bach2 KO mice was partially attenuated in the Treg cell-infused mice (Supplementary Fig. 2b), confirming that the transferred Treg cells were functional. Nevertheless, serum levels of IgM and IgG Abs against dsDNA did not differ significantly between the Treg cell recipients and vehicle recipients at 4 and 8 weeks post-infusion (Fig. 2f), and renal deposition of IgM and IgG was also unaffected (Fig. 2g). These results indicate that the IgG autoantibody-mediated pathology associated with Bach2 deficiency is not simply due to deficiency in Treg cells.

### Transcriptome differences between Bach2-deficient and WT B cells

**Fig. 3:**
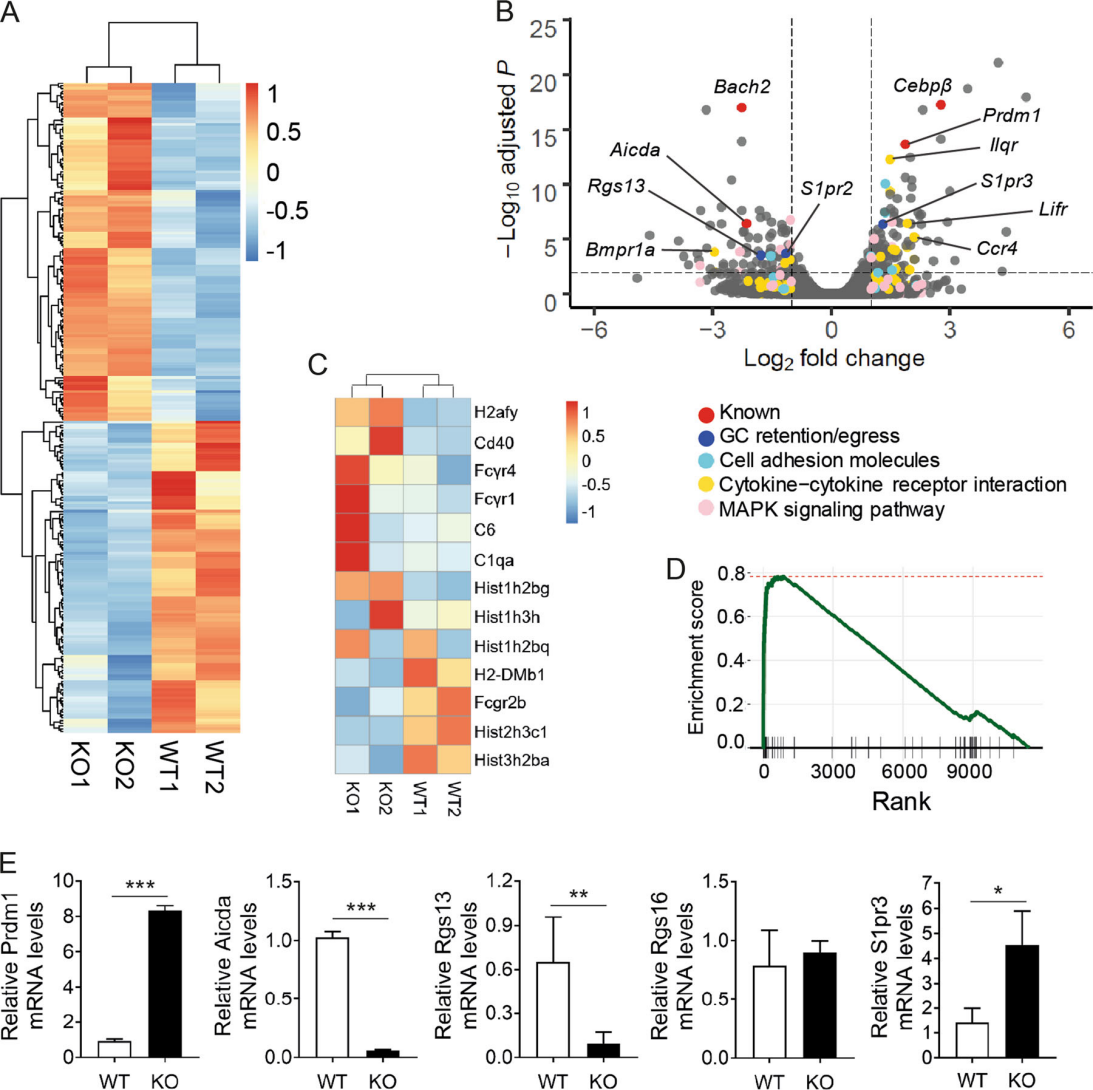
Transcriptome analysis of Bach2-deficient and control B cells. **a** Heatmap of gene expression changes between Bach2 KO and WT B cells. **b** Volcano plot of gene expression changes between Bach2 KO and WT B cells. Genes involved in KEGG pathways are shown as indicated. **c** and **d** Heatmap and enrichment plot of genes in the KEGG pathway of SLE. **e** Quantitative RT-PCR to verify the RNAseq data. Graphs are representative of two independent experiments. *p < 0.05, **p < 0.01 and ***p < 0.001 by Student’s *t*-test.

To identify genes and pathways involved in EF activation in Bach2-deficient B cells, we carried out RNAseq analyses of splenic CD19^+^ B cells from Bach2 KO and WT mice at ~20 weeks of age. These results revealed a different pattern of gene expression in the two cell types. A total of 234 genes were differentially expressed between them, with 122 genes upregulated and 112 genes downregulated, according to the DEG criteria we established (Fig. 3a, b). The upregulation of *Prdm1* and *Cebpb* and the downregulation of *Aicda*, which was known previously^21,35^, verified the status of the cells used to obtain the RNAseq data. Interestingly, genes related to GC confinement of B cells (*Rgs13 and S1pr2*)^36,37^ were downregulated, whereas a gene related to GC egress of B cells (*S1pr3*)^37^ was upregulated (Fig. 3b, e), suggesting a B cell-autonomous function of Bach2 in GC formation. The result that expression of *Rgs16*, another gene related to GC confinement of B cells, was not altered by the absence of Bach2 suggested that downregulation of *Rgs13* relied on the function of Bach2 but not the consequence of altered GC formation.

We next identified enriched pathways among the 186 KEGG pathways to identify those that specifically guide the physiology of Bach2-deficient B cells. Surprisingly, the pathways with particular immunologic significance that were differentially enriched included “KEGG of SLE” (adjusted *P* = 0.046 by Benjamini–Hochberg methods) (Fig. 3c, d). This result suggests that SLE-related genes are more highly activated in Bach2-deficient B cells than in WT B cells.

### B cell-specific deletion of Bach2 is sufficient to impair the development of GC B cells but not to drive EF Ab responses

**Fig. 4:**
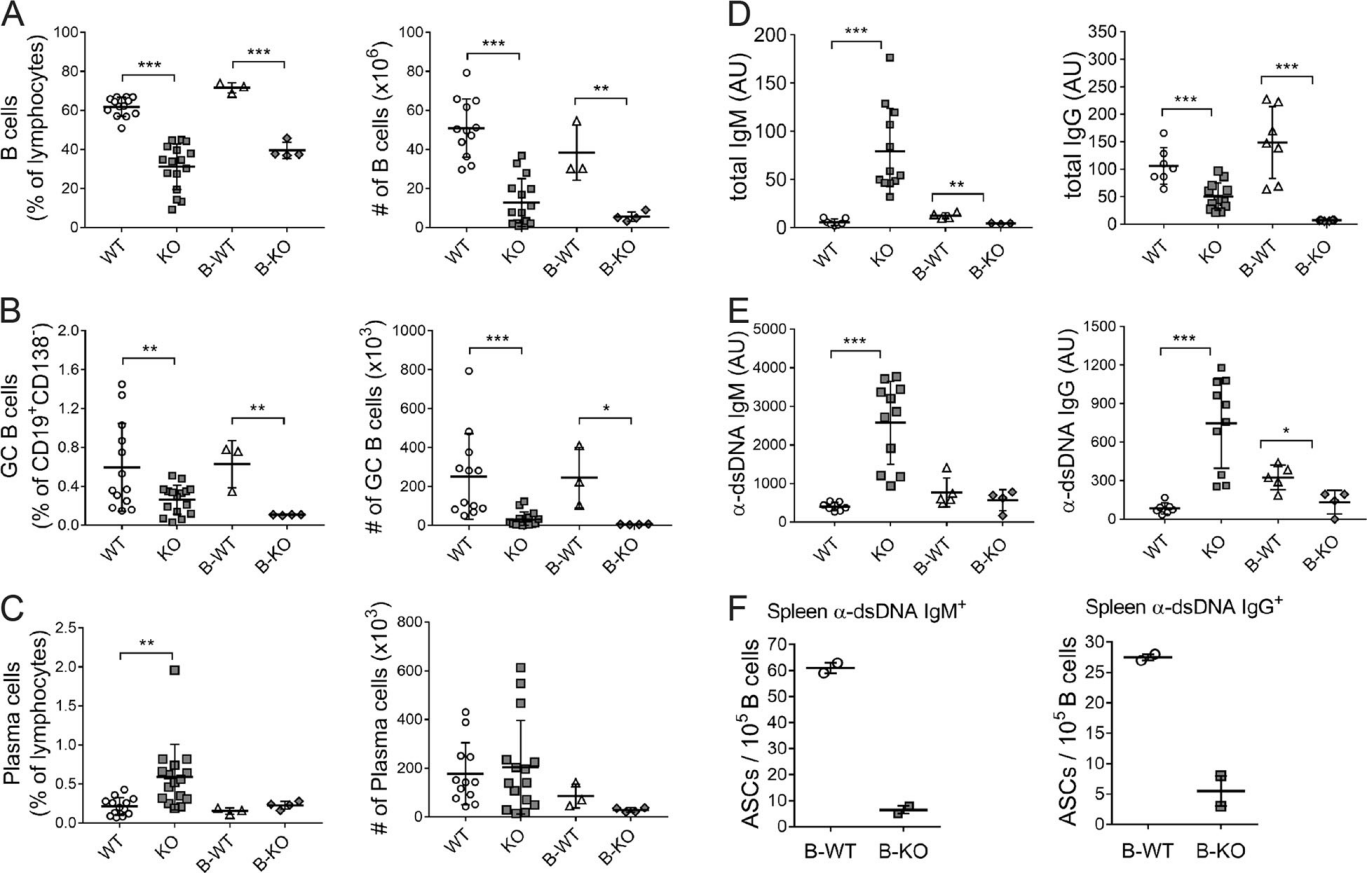
B cell-autonomous functions of Bach2. *Rag2*^−/−^ mice were reconstituted with μMT BM cells plus either Bach2 KO or WT BM cells to generate B-KO and B-WT chimeric mice. Fifteen weeks after BM reconstitution, sera and spleen cells were harvested from the recipients and assayed by FACS **a**–**c**, ELISA **d**, **e** and ELISPOT **f**. FACS profiles gated on CD19^+^CD138^-^ cells and live lymphocytes are used to enumerate FAS^+^GL7^+^ GC B cells and CD19^-^CD138^+^ plasma cells, respectively **b**, **c**. The data shown were pooled from three independent experiments. **a**–**e** or are representative of two independent experiments **f** and are displayed as the means ± SEMs, with symbols representing the values of individual mice. *p < 0.05, **p < 0.01 and ***p < 0.001 by Student’s *t*-test.

We asked whether the GC-independent production of IgG autoantibodies seen in Bach2 KO mice stemmed from B cell-autonomous mechanisms. To address this question, we generated BM chimeric mice by transferring a mixture of μMT BM cells and Bach2 KO BM cells into *Rag2*^*−/−*^ mice (referred to hereafter as B-KO) and assayed them *ex vivo* at 15 weeks post-reconstitution. A mixture of μMT BM cells plus WT BM cells served as a control (referred to hereafter as B-WT). Similar to KO mice, the B-KO mice had reduced proportions and numbers of CD19^+^ B cells and GC B cells (Fig. 4a, b). However, unlike the KO mice, the B-KO mice did not have a higher proportion of CD19^lo^CD138^+^ plasma cells than their B-WT counterparts (Fig. 4c). Consistent with this result, the serum titers of total and dsDNA-specific IgM Abs were not elevated in the B-KO mice, and those of IgG Abs were significantly reduced (Fig. 4d, e). Cells secreting IgM and IgG anti-dsDNA Abs were also less numerous in the spleens of the B-KO mice than in those of B-WT mice (Fig. 4f). These results demonstrate that B cell-intrinsic loss of Bach2 is sufficient to cause B lymphopenia and impaired development of GC B cells but insufficient to drive EF Ab production.

### T cell-specific deletion of Bach2 induces CD4^+^ T cells to spontaneously differentiate into Tefh cells

**Fig. 5:**
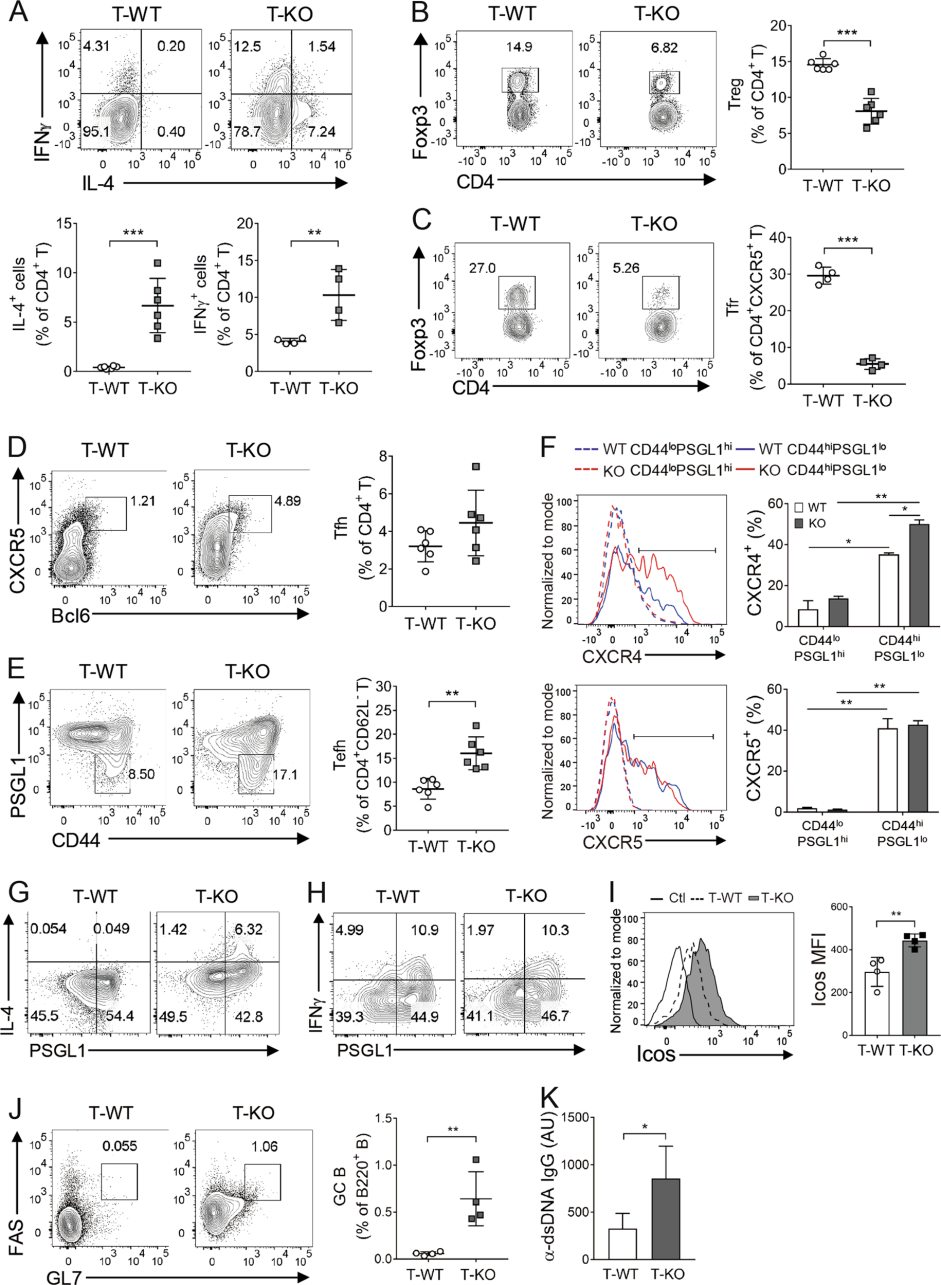
T cell-autonomous functions of Bach2. *Rag2*^*−/−*^ mice were reconstituted with *Tcrβ*^*−/−*^ BM cells and either Bach2 KO or WT BM cells to generate T-KO and T-WT chimeric mice. Approximately 10 weeks after BM reconstitution, spleen cells and sera were collected from the recipients and assayed by FACS **a**–**e** and **g**–**j** and ELISA **k**, respectively. Spleen cells from approximately 20-week-old Bach2 KO mice and their WT littermates were assayed by FACS **f**. Representative FACS profiles gated on CD4^+^ **a**, **b** and **d**, CD4^+^CXCR5^+^ **c**, CD4^+^CD62L^−^ **e**–**h**, CD4^+^CD62L^−^CD44^hi^PSGL1^lo^ **i**, and B220^+^ **j** are shown. Data are representative of more than three independent experiments and are displayed as the means ± SEMs, with or without symbols representing the values of individual mice. AUs, arbitrary units; Ctl, negative control staining. *p < 0.05, **p < 0.01 and ***p < 0.001 by Student’s *t*-test.

Because the above data imply that non-B cell-intrinsic loss of Bach2 is required for EF production of IgG autoantibodies by Bach2-deficient B cells, we extended our experiments to address whether Bach2 deficiency induces the spontaneous development of Tefh cells in a cell-autonomous manner. To this end, we generated another type of mixed BM chimeric mouse using *Tcrβ*^−/−^ BM cells instead of the μMT BM cells used in Fig. 4 and assayed Th subsets arising spontaneously approximately 10 weeks after reconstitution. As expected, the chimeric mice reconstituted with *Tcrβ*^*−/−*^ BM and Bach2 KO BM cells (referred to as T-KO) contained higher proportions of Th2 and Th1 cells and a lower proportion of Treg cells than the control chimeric mice reconstituted with *Tcrβ*^*−/*^^−^ BM and WT BM cells (referred to as T-WT) (Fig. 5a, b). Th17 cells were almost absent in both types of mice (data not shown). In the T-KO mice, the proportion of Foxp3^+^ cells among CD4^+^CXCR5^+^ cells, which represent follicular regulatory T (Tfr) cells, was also reduced, while that of CD4^+^CXCR5^+^BCL6^+^ Tfh cells was not significantly altered, resulting in an enhanced ratio of Tfh cells to Tfr cells (Fig. 5c, d). Furthermore, the T-KO mice contained a higher proportion of CD4^+^CD62L^−^CD44^hi^PSGL1^lo^ cells than the T-WT mice (Fig. 5e). Aged whole KO mice also had an increased fraction of the same phenotype cells relative to that of their WT counterparts, and this fraction exclusively contained both CXCR4^+^ cells and CXCR5^+^ cells (Fig. 5f). Importantly, the percentage of CXCR4^+^ cells within the fraction was significantly elevated in KO mice and that of CXCR5^+^ cells within the same fraction was not altered. These data indicated that the fraction of cells with CD4^+^CD62L^-^CD44^hi^PSGL1^lo^ phenotypes contained heterogeneous populations, including Tefh and Tfh cells, and that the proportion of Tefh cells was higher in T-KO mice than in T-WT mice. In support of this finding, the cells within the fraction were not a major source of IL-4 or IFN-γ, distinctive from Th2 and Th1 cells (Fig. 5g, h). IL-21 expression was not detected in any fractions (not shown). Interestingly, the CD4^+^CD62L^−^CD44^hi^PSGL1^lo^ cell fraction from the T-KO mice expressed *Icos* at a level higher than that from T-WT mice (Fig. 5i). Finally, we found that GC B cells and IgG anti-dsDNA Ab were significantly elevated in the T-KO mice (Fig. 5j, k). These results demonstrate that the absence of Bach2 favors the spontaneous differentiation of CD4^+^ T cells into Tfh and Tefh cells in a cell-autonomous manner, leading to enhanced Ab responses via GC-mediated and EF routes, respectively.

### Bach2 represses *Icos* transcription in CD4^+^ T cells

**Fig. 6:**
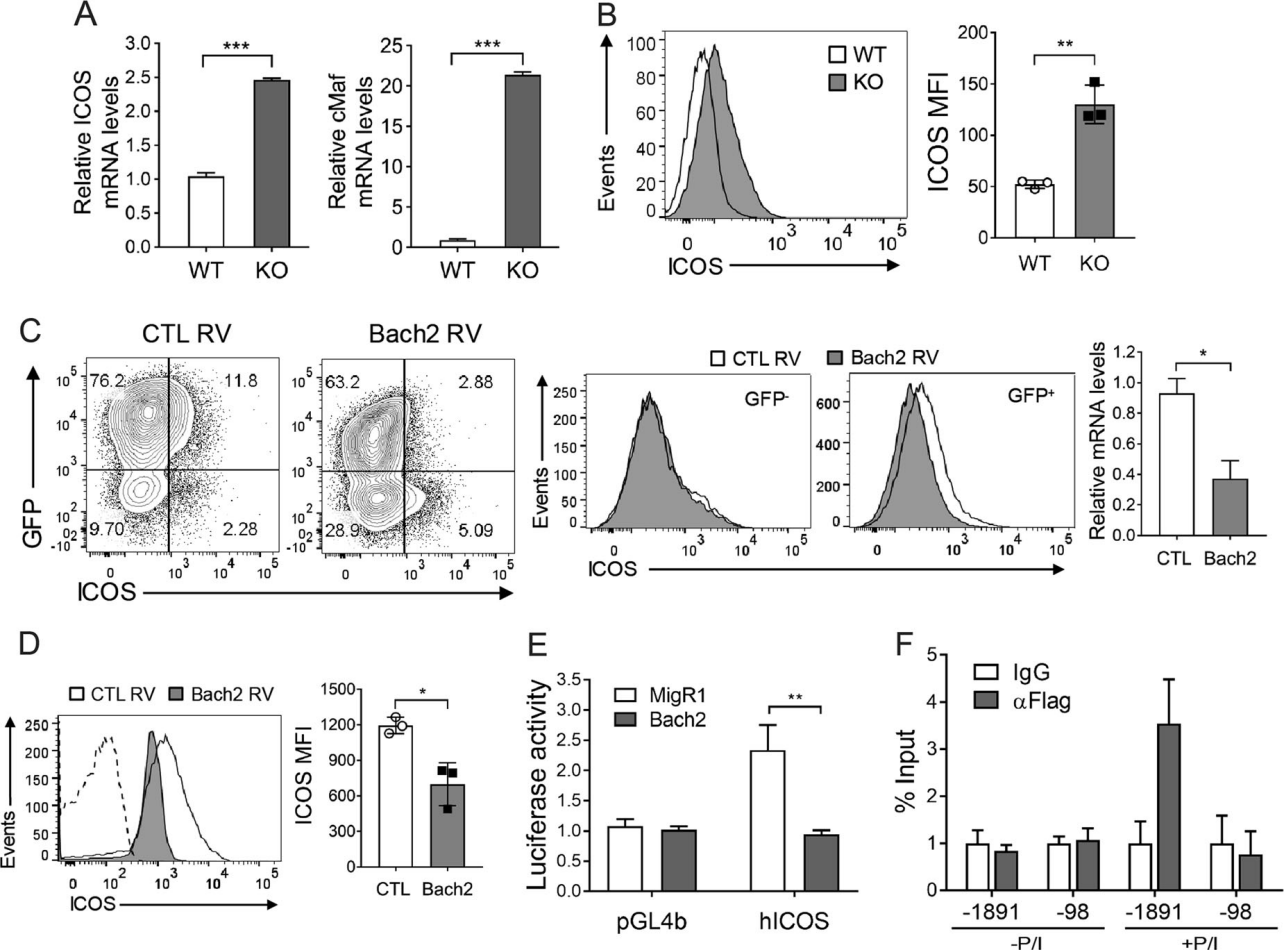
Bach2 represses Icos transcription. **a**, **b** Splenic CD4^+^ T cells from Bach2 KO and WT mice were assayed by quantitative RT-PCR **a** and FACS **b**. **c**, **d** CD4^+^ T cells infected with either a Bach2- or an empty vector-expressing retrovirus (RV) were assayed by FACS and RT-PCR **c** and transferred to *Tcrβ*^−*/−*^ mice, followed by FACS analysis 10 days later **d**. FACS profiles gated on CD4^+^ cells are shown. **e** Jurkat T cells were cotransfected with a pGL4b-hICOS reporter and a MigR1–Bach2 or an empty vector, stimulated with PMA and ionomycin, and assessed by a luciferase activity assay. **f** Jurkat T cells were transfected with the pcDNA3-2 × FLAG-Bach2 vector, incubated in the presence or absence of PMA and ionomycin (P/I), and assayed by ChIP-qPCR. All data are representative of three independent experiments. *p < 0.05, **p < 0.01 and ***p < 0.001 by Student’s *t*-test.

The aforementioned data led us to hypothesize that Bach2 acts as a repressor of factor(s) that regulate(s) the pathways of Tefh cell development. We focused on the costimulatory molecule Icos as the putative regulator because Icos is required for Tefh cell development^10^, and we noted MARE-like sequences as potential Bach2-binding sites −1891 to −1899 and −98 to −108 bp upstream of the human *ICOS* transcription start site via *in silico* analysis. These sequences had >77% sequence homology with the canonical MARE sequence. Indeed, we found that when stimulated via TCR and CD28, Bach2-deficient CD4^+^ T cells produced higher levels of *Icos* mRNA and cell-surface Icos than control CD4^+^ T cells (Fig. 6a, b). c-Maf, a downstream signaling molecule in the Icos pathway, was also more abundant in the Bach2 KO cells than in WT cells. Conversely, CD4^+^ T cells transduced with a Bach2-expressing retrovirus downregulated Icos expression on the cell surface and at the mRNA level (Fig. 6c). To confirm this function of Bach2 in vivo, we transduced CD4^+^ T cells with either a Bach2- or an empty vector-expressing retrovirus and adoptively transferred the cells into *Tcrβ*^*−/−*^ mice. The CD4^+^ T cells transduced with the Bach2-expressing retrovirus had significantly lower levels of surface Icos than those transduced with empty vector (Fig. 6d). These results reveal an inverse correlation between the levels of Bach2 and Icos.

To test the hypothesis that Bach2 binds to the *ICOS* promoter and represses its transcription, we carried out luciferase reporter assays with a reporter construct containing 2000 bp of the human *ICOS* promoter upstream of the transcription start site. Enforced expression of Bach2 reduced the induction of luciferase expression, indicating that Bach2 inhibits activation of the *ICOS* promoter (Fig. 6e). This result supports the idea that Bach2-binding sites are present in the 2000 bp region upstream of the *ICOS* transcription start site. In ChIP-qPCR assays, immunoprecipitation of cross-linked chromatin with Abs to FLAG-tagged Bach2 but not with IgG enriched for genomic regions containing the −1891 to −1899 MARE-like sequences in stimulated cells, but the −98 to −108 region was not enriched (Fig. 6f). This result shows that Bach2 binds to the distal MARE-like site of the *ICOS* promoter but not to the proximal site.

## Discussion

We demonstrate here that the effects of Bach2 deficiency on Ab responses differ depending on the immunologic context. *Bach2*^*−/−*^ mice exhibit defects in GC formation and whole IgG production in the steady state and upon deliberate immunization. However, despite a lack of GCs, these mice produce increased levels of IgG autoantibodies accompanied by histopathologic manifestations akin to lupus nephritis that are not dependent on Treg cell deficiency. The defect in GC formation is B cell-autonomous, and this effect is not sufficient to induce EF development of postswitched plasma cells. On the other hand, T cell-specific deletion of Bach2 favors the spontaneous development of Tefh cells, and this effect is associated with the role of Bach2 as a repressor of *Icos*. Taken together, our results show that Bach2-deficient autoreactive B cells undergo IgG CSR outside the GC and that this process requires Bach2-deficient CD4^+^ T cells that preferentially differentiate into humoral effectors. Therefore, it seems that the cell-autonomous activities of Bach2 in B cells and in cognate CD4^+^ T cells are required to maintain self-tolerance.

It is puzzling that Bach2-deficient self-reactive B cells can undergo CSR through the EF pathway even though Bach2 is required for CSR and GC formation. This finding may mean that Bach2 is dispensable for CSR at EF sites and essential in GC reactions. We suspect that this contradiction stems at least in part from the different kinetics of EF and GC reactions. GC B cells cycle several times between light and dark zones to achieve CSR and other maturation processes and ultimately exit the GC as plasma cells secreting high-affinity class-switched Abs^8,38^. Therefore, a mechanism must exist that secures a time window for the action of AID and prevents the accidental exit of immature plasma cells during the GC reaction. Bach2 seems to provide this mechanism by repressing the expression of Blimp-1 instead of directly repressing *Aicda*^22^. In contrast, because EF B cells do not shuttle between zones and differentiate into plasma cells more rapidly than GC B cells, Bach2 may not be required for executing CSR during the EF response.

Normal self-reactive B cells tend to lose their self-reactivity in GCs through self-tolerance mechanisms, including negative selection, Ab redemption, and receptor re-editing^39–41^. Therefore, some kind of process by which self-reactive B cells evade the GC checkpoint is needed for them to maintain their autoimmunity. Our finding that Bach2-deficient self-reactive B cells not only fail to form GCs but are also able to evolve to pathogenic plasma cells through the EF pathway suggests that downregulation of Bach2 is one mechanism by which autoreactive B cells override the GC checkpoint and maintain their self-reactivity.

Our finding that the cell-autonomous activity of Bach2 is not sufficient to allow the accumulation of IgG autoantibody-secreting EF plasma cells underscores the importance of Bach2 activity in B cell helpers. Indeed, we found that T cell-specific ablation of Bach2 could induce CD4^+^ T cells to differentiate spontaneously into Tefh cells as well as Th2 cells and enhance the ratio of Tfh to Tfr cells. We speculate that Tefh cells are the most important subset in promoting the maturation of Bach2-deficient autoreactive B cells for several reasons. First, since Bach2-deficient mice have a B cell-autonomous defect in GC formation, Tfh cells would be without effect. Second, Th2 development is considered dispensable for IgG (even IgG_1_) production, given the location of Ab responses and the normal production of IgG isotypes seen in Th2-deficient mice^42^. These reasons collectively point to the importance of Tefh cells in humoral autoimmune responses in Bach2-deficient mice. In this regard, it is noteworthy that we identified for the first time Bach2 as a regulator of Tefh cells.

We found that induced immunity and autoimmunity are differentially affected by Bach2 deficiency. The latter appears to be relatively unaffected by the absence of Bach2 in terms of IgG Ab production. This finding may mean that T cell-dependent Ab responses occurring in response to active immunization are more dependent on the GC reaction than autoantibody production and that the EF event plays a more important role in autoimmunity than in active immunization. Previous reports have provided evidence that the requirements of these two processes differ. One example is that IFNγR is needed for GC formation in spontaneous autoimmunity but not in active immunization^32,33^. Our data provide another example of such different requirements, with Bach2 needed for the latter but not the former.

Bach2 has been shown to play a critical role in cell fate choice at diverse lineage bifurcation points. In the absence of Bach2, common lymphoid progenitors and common myeloid progenitors give rise to myeloid cells rather than B cells and erythroid cells, respectively^43,44^. At the pre-B cell checkpoint, Bach2-deficient pre-B cells are positively selected, while control pre-B cells meet the opposite fate^45^. Our evidence for a preferential effect of the EF reaction over the GC reaction points to an additional bifurcation point where Bach2 plays a role in cell fate choice.

How can Bach2 achieve this effect? The underlying mechanism remains largely unknown, but our transcriptome analysis of genes related to GC retention and egress may provide one clue to the answer. Regulator of G protein signaling (Rgs)13 and Rgs16 are highly expressed in GC B cells and accelerate the intrinsic rate of Gα GTPase activity linked to CXCR4 and CXCR5, respectively, leading to prolonged retention of B cells in the GC^37,46,47^. This arrest of migration may stabilize GCs, thereby providing an optimal microenvironment for the production of pathogenic autoantibodies. Moreover, a loss-of-function mutation of *Rgs13* was found to promote EF plasma cell production and perturb the normal GC B gene expression program^48^. These findings are in agreement with our data showing downregulation of Rgs13 in Bach2 KO B cells. In addition to Rgs13, the elevated ratio of sphingosine 1 phosphate receptor (S1PR)3 to S1PR2 in these cells may contribute to their defect in GC formation, given that S1PR3 and S1PR2 are known to act in the GC dissemination and confinement of B cells, respectively^37^. Thus, we propose that the absence of Bach2 early in activated B cells results in a loss of Rgs13 and an elevated ratio of S1PR3 to S1PR2 and finally leads to a failure of GC formation.

In conclusion, we demonstrate here that Bach2 is a genetic risk allele for SLE in mice. Autoreactive Bach2-deficient B cells escape from the GC checkpoint for self-tolerance and cooperate with Bach2-deficient Th cells to produce IgG class-switched pathogenic autoantibodies at the EF site. Thus, our study provides insights into the origin of the humoral autoimmunity accompanying SLE.

## Supplementary information

Supplemental materials
