# Supplementary material for: Bach2 deficiency leads autoreactive B cells to produce IgG autoantibodies and induce lupus through a T cell-dependent extrafollicular pathway
Source: Exp Mol Med. 2019 Dec 9;51(12):148. doi: 10.1038/s12276-019-0352-x (PMC6901549; doi:10.1038/s12276-019-0352-x)
Supplement: Supplementary file 1 — Supplemental materials [file 12276_2019_352_MOESM1_ESM.pdf]

Supplementary Table 1. Monoclonal Abs used for FACS

Abs to	Clones	Manufacturers
CD4	RM4-5	BD Biosciences
Fas	Jo2	BD Biosciences
B220	RA3-6B2	BD Biosciences
CD138	281-2	BD Biosciences
CXCR4	2B11	BD Biosciences
CXCR5	2G8	BD Biosciences
Bcl6	K112-91	BD Biosciences
PSGL1	2PH1	BD Biosciences
CD62L	MEL-14	BD Biosciences
GL7	GL7	eBioscience
Foxp3	FJK-16s	eBioscience
IL-4	BVD6-24G2	eBioscience
IFN-γ	SMG1.2	eBioscience
CD44	IM7	BioLegend
ICOS	REA192	Miltenyi Biotec

Supplementary Table 2. The primer sequences used for quantitative PCR

Genes or loci	Directions	Sequences
β-actin	Forward	5'-GACGGCCAGGTCATCACTATTG-3'
	Backward	5'-AGGAAGGCTGGAAAAGAGCC-3'
Prdm1	Forward	5’-TTCTTGTGTGGTATTGTCGGGACTT-3’
	Backward	5’-TTGGGGACACTCTTTGGGTAGAGTT-3’
Aicda	Forward	5’-TTCAACAGCATGGAAAAGCA-3’
	Backward	5’-TGGCTTGTGATTGCTCAGAC-3’
Rgs13	Forward	5’-TCTACATCCAGCCACAGTCTCCTA-3’
	Backward	5’-CACGAATGCTTTTGATGATAGCTT-3’
Rgs16	Forward	5’-CCGGCCTGTGAGCCCCTTTC-3’
	Backward	5’-GGAGCGCAGGGCCCAGTAAG-3’
S1pr2	Forward	5’-AAATCCAATACGGTACAAACG-3’
	Backward	5’-GGTCAGACAGCACCCACA-3’
S1pr3	Forward	5’-TCAGGGAGGGCAGTATGTTC-3’
	Backward	5’-GAGTAGAGGGGCAGGATGGT-3’
Icos	Forward	5’-CTCACCAAGACCAAGGGAAGC-3’
	Backward	5’-CCACAACGAAAGCTGCACACC-3’
cMaf	Forward	5’-GCAGAGACACGTCCTGGAGTCG-3’
	Backward	5’-CGAGCTTGGCCCTGCAACTAGC-3’
The distal MARE-like	Forward	5'-CTCTGTGACTCCGGGCAAAT-3'
	Backward	5'-ACAACTCAGCGAGGGAGAGTCT-3'
The proximal MARE-like	Forward	5'-GGTAATGTTTTCCTCTGTCTTCAAAAT-3'
	Backward	5'–TGTTTCTTCTTTGATGCCAATGG-3'
CYCLOPHILIN	Forward	5'-TGC CATCGCCAAGGAGTAG-3'
	Backward	5'-TGCACAGACGGTCACTCAAA-3'

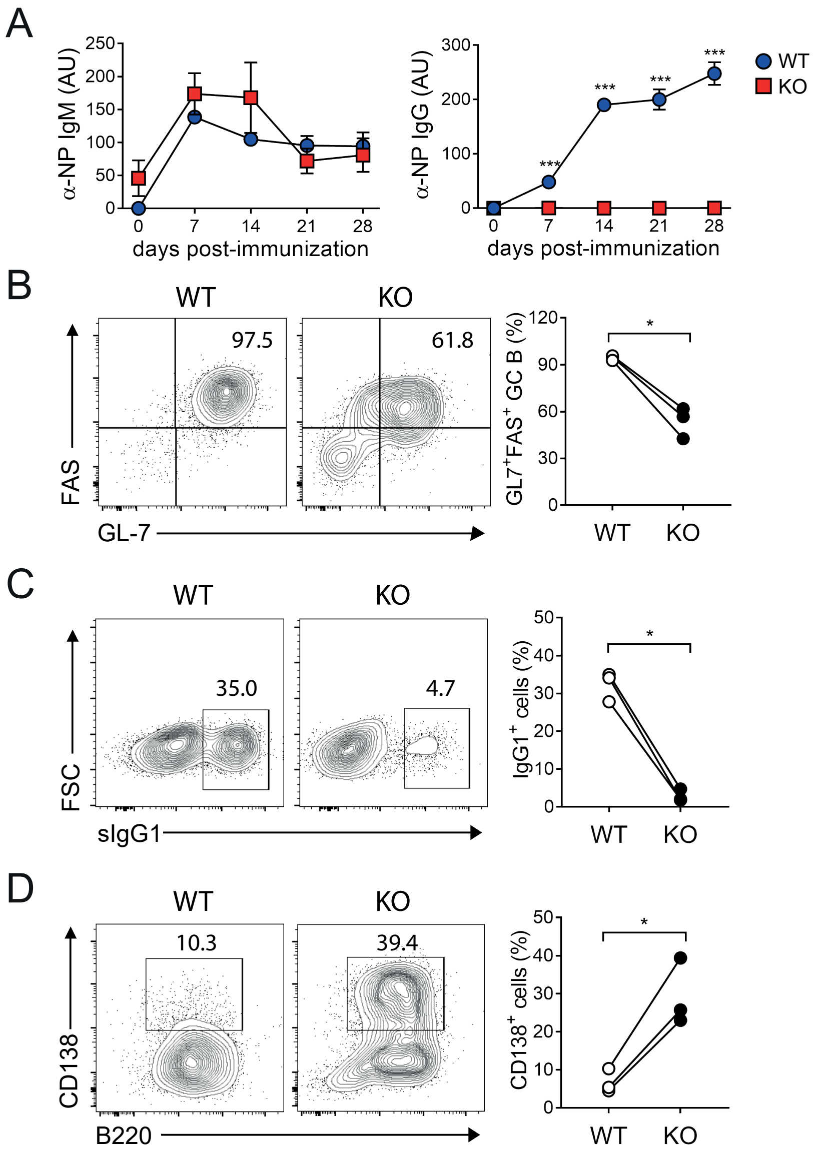

Supplementary Figure 1. Bach2-deficient B cells are defective in becoming germinal center B cells and undergoing class switch recombination upon active immunization and in vitro T-dependent stimulation. A, Approximately 8-week-old Bach2 knockout (KO) mice and their wildtype (WT) littermates were injected with NP-KLH plus alum. Sera were collected at the indicated times and assayed by ELISA. Data are presented as means ± SEM (n=3-5 mice/group) and are representative of three independent experiments. **B-D,** Splenic B220$^+$ cells from Bach2 KO mice and their WT littermates were sorted by negative selection, cultured in the presence of CD40L and IL-4 for 4 days, and analyzed by FACS. Representative FACS profiles gated on B220$^+$CD138$^-$ cells (B) and B220$^+$ cells (C and D) with cell percentages in the indicated areas. The graphs show the results of three independent experiments. Data were analyzed by two-tailed paired Student's *t*-test. **p* <0.05, ***p* <0.01, ****p* <0.001.

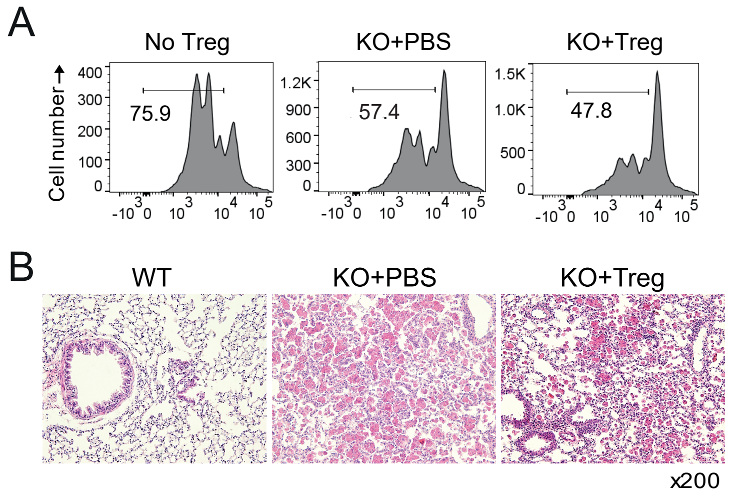

Supplementary Figure 2. The effect of regulatory T cell reconstitution in Bach2-deficient mice. Bach2 knockout (KO) and wildtype (WT) mice at 8-12 weeks of age were infused intravenously with normal syngeneic Foxp3$^+$ regulatory T (Treg) cells or PBS as vehicle control. Eight weeks after infusion the recipients were assayed post mortem. **A,** CD4$^+$CD25^{hi} Treg cells were sorted from PBS recipients (KO+PBS) and Treg cell recipients (KO+Treg) 8 weeks after infusion, and cocultured with WT CD4$^+$ T cells labeled with 3 μM carboxyfluorescein succinimidyl ester (CFSE; Molecular Probes) in the presence of anti-CD3 and anti-CD28 Abs (BD Biosciences) for 72 h, followed by flow cytometry. **B,** Lung tissues from the recipients were examined by histopathologic methods. Representative images of 2 mice per group are shown.
